# The Effect of Cold Treatment of Parboiled Rice with Lowered Glycaemic Potency on Consumer Liking and Acceptability †

**DOI:** 10.3390/foods7120207

**Published:** 2018-12-16

**Authors:** Louise Weiwei Lu, John Monro, Jun Lu, Elaine Rush

**Affiliations:** 1 Human Nutrition Unit (HNU), School of Biological Sciences, University of Auckland, Auckland 1024, New Zealand; 2 School of Sport and Recreation, Faculty of Health and Environmental Sciences, Auckland University of Technology, Auckland 1010, New Zealand; elaine.rush@aut.ac.nz; 3 The New Zealand Institute for Plant & Food Research, Palmerston North 4474, New Zealand; john.monro@plantandfood.co.nz; 4 School of Science, and School of Interprofessional Health Studies, Faculty of Health and Environmental Sciences, Auckland University of Technology, Auckland 1010, New Zealand; jun.lu@aut.ac.nz; 5 College of Life Sciences, Shenzhen University, Shenzhen 518060, China

**Keywords:** parboiled rice, medium-grain white rice, medium-grain brown rice, sensory evaluation, consumer acceptability

## Abstract

A significant reduction in rice starch digestibility and subsequent postprandial blood glucose responses following extended cold treatment (at 4 °C for 24 h) have been demonstrated in both in vitro and in vivo studies, respectively. The impact of cold treatment was more significant for parboiled rice compared to other rice varieties. This study aimed to investigate consumer liking of sensory characteristics that may influence consumer acceptability of three available rice products in the Auckland region (medium grain white, medium grain brown and parboiled rice, which were either freshly boiled or cold-treated and reheated). The consumer liking of sensory characteristics (colour, taste, flavour, and texture) of each rice sample were accessed using visual analogue scales (VAS) in a randomized single blind setting. In the second stage, the participants evaluated their acceptability on VAS after the nutritional value and the characteristics of the rice samples were revealed. Sixty-four rice consumers reported higher likings of sensory characteristics of cold-treated parboiled rice and medium grain brown rice. The effect of cold treatment on the liking of sensory characteristics was more significant for parboiled rice (*p* < 0.05). Participants who are between 36 and 55 years old and consume rice domestically more than 10 times per month preferred cold-treated brown rice (73.8% of the participants’ population (67.4%, 80.2%)) and parboiled rice (74.3% of the participants’ population (67.9%, 80.7%)) (*p* < 0.001). As a result, cold-treated reheated parboiled rice received higher likings and acceptability and could be recommended and accepted as a healthier replacement of the daily staple meal.

## 1. Introduction

Rice is a widely consumed staple food, but there is wide variation in the rice products consumed, depending on the cultivar, processing technologies (refining rice by removing the husk and bran), and pre-cooking treatment (e.g., parboiling). Previous laboratory experiments on in vitro rice starch digestion [1], and human participant’s glycaemic responses to freshly cooked and cold-treated reheated rice samples [2], reported that rice parboiled, cooked and cold treated for 24 h attenuated rice starch digestibility, extended chewing time, decreased postprandial glycaemic responses and had improved palatability compared to white and brown rice. This evidence supports advice that substituting parboiled rice for medium grain white rice products, which are commonly consumed by rice consumers, and practicing the safe prolonged cold treatment method may improve postprandial blood glucose response, reduce glycaemic load and benefit the control of long-term glycaemia among rice consumers.

Reported physico-chemical differences among cooked rice meals may be considered to be an important attribute to different sensory properties that may influence rice consumers’ choices. Parboiled rice is steam treated paddy rice that has a yellow-tinted colour, a firm and chewy texture, and a distinct strong flavour. In comparison, white rice has a pale white colour, a soft and adhesive texture, and a light and starchy flavour [3,4]. Previous studies reported that these distinct characteristics of parboiled rice were disliked by some rice consumers from particular regions, such as East and Southeast Asia [5,6], but liked by those from regions such as India, Pakistan, Brazil and Ghana [4,5,7]. Moreover, various post-cooking methods (including cooling, cold storage, and reheating) may also influence the physical properties and the liking of sensory properties of cooked rice [8]. 

Rapidly changing demographics, including an aging population, lifestyle changes that are related to increasing cultural diversity and infiltration, and the consumption shift towards convenience food or pre-cooked ready-to-eat food products, might have impacted the consumer’s choices for food, in terms of liking and acceptability [9,10]. Auckland, New Zealand has an ethnically diverse population which is undergoing a transformation. As the liking and acceptability to consumers may differ among different ethnicities, age groups, and socio-economic groups, these demographic factors present challenges when a functional food or foods with health benefits are introduced to the consumer population. The present study proposed to better understand the consumer liking and acceptability of rice prepared in different ways: Freshly cooked or prolonged cold-treated and reheated. As consumers evaluate food quality predominantly based on both the sensory and nutritional characteristics [11], an unblinded consumer acceptability test would assist with the recommendations of the nutritional values of rice products as a staple food and rice cooking and preparation method.

This study aimed to evaluate whether prolonged cold-treated cooked parboiled rice with a slower glycaemic release would be liked and accepted as a healthier replacement by Auckland consumers who commonly consume either freshly boiled plain medium-grain white or brown rice as their staple grain. The study investigated the following questions: (1) Would consumers report a significantly different liking of the sensory properties (colour, texture, flavour and taste) of reheated parboiled rice compared to freshly cooked or cold-treated medium-grain white rice and medium-grain brown rice, and freshly cooked parboiled rice? (2) Would cold-treated parboiled rice be acceptable to consumers as a replacement to the rice products they currently consume when the relative importance of healthiness is revealed as a favourable feature?

## 2. Materials and Methods 

### 2.1. Rice Products

Three rice products were selected for the study and their characteristics were as follows:

- Australian imported raw medium-grain white rice (SunRice^®^, Sydney, Australia), which is widely available in Auckland, New Zealand. It was selected as the most commonly consumed staple rice and the control sample.
- Australian imported raw medium-grain brown rice (SunRice^®^, Sydney, Australia), which is widely available in Auckland, New Zealand. It was selected as a healthier alternative to medium grain white rice.
- Parboiled rice, produced and imported from Thailand (RealRice^®^, Auckland, Thailand imported). It was selected as the healthiest alternative based on the results from a previous in vitro study on rice starch digestibility and glucose release [1].

The medium-grain white and medium-grain brown rice were characterised as medium-grain commercial rice (*Oryza sativa* L.) [12], and cultivated and processed in Riverina, Australia, in 2013. The parboiled long-grain rice was cultivated in Thailand and harvested and processed in late 2012 and 2013.

Six samples of rice were prepared: Freshly cooked medium-grain white rice, freshly cooked medium-grain brown rice, freshly cooked parboiled rice, reheated medium-grain white rice, reheated medium-grain brown rice, and reheated parboiled rice.

### 2.2. Cooking Method

Cooking, storing, and reheating methods were as described for previous studies [1,2]. The quantities of rice, water added, and times of cooking were as recommended by the manufacturer. The temperature of cooking (100 °C) and reheating (65 °C) were monitored and the room temperature (23 °C) and humidity (35%) of the cooking environment remained stable. Three rice products were cooked in three separate 3 L domestic automated commercial rice cookers (Abode^®^ Rice Cooker, BIGW_7963940) following the instructions provided by the rice product manufacturers. To achieve full gelatinization (i.e., until automatic completion in the rice cooker), the rice to water ratio were different for each rice product: 1 measuring cup of rice (141.9 ± 5.0 g) to 1½ cups of water (375 mL) for medium-grain white rice were cooked for approximately 20 min; 1 cup of rice (130.8 ± 5.0 g) to 2 cups of water (500 mL) for medium-grain brown rice were cooked for approximately 25 min; and, 1 cup of rice (135.3 ± 5.0 g) to 2⅓ cups of water (583.3 mL) for parboiled rice were cooked for approximately 30 min. All freshly cooked rice was maintained in a sealed warm container at 65 °C until served.

### 2.3. Storing and Reheating Method

The freshly cooked rice samples were spread evenly in a shallow plastic pan (20 cm × 20 cm) and sealed with food wrap (30 cm × 30 cm) to prevent moisture loss and for food safety purposes. All samples were transferred to the refrigerator within 30 min for rapid and even cooling to 4 °C. The rice samples were kept in the refrigerator (at a stable temperature of 4 °C) for 24 h. After 24 h, the temperature of the rice was checked again and the rice samples were reheated in the microwave, mixed thoroughly, and the temperature checked several times until they reached 65 °C. All reheated rice products were kept at 65 °C until served.

### 2.4. Participants

Volunteer consumers were recruited at Auckland North Shore Akoranga area (including Auckland University of Technology (AUT) North Campus and surrounding area) and Auckland City (including AUT City Campus and surrounding area) between January and March 2015. Volunteers were screened by questionnaire to confirm that they met the inclusion criteria of being in general good health; 18 to 80 years old; were regular rice consumers (consuming plain cooked rice at least once per week for the previous year and intending to consume rice as a staple food in the future); and had consented to complete the entire tasting and rating session (three freshly cooked and three reheated rice samples). Exclusion criteria included health issues (e.g., diabetes, cardiovascular diseases, cancer, and/or major surgery), known allergies, and difficulties in perceiving smell and taste of foods or swallowing foods. All participants were asked to fast for at least two hours before participating in the study. A sample size of over 60 was required to detect a difference of 14.8% between rice treatments based on *F*-test (ANOVA repeated measures) with an alpha value of 0.05, and a beta value of 0.10 [13].

This study was approved by the AUT Ethics Committee (AUTEC) (Reference: 13/183 Which rice and why?). The flow chart of the experimental design of the consumer liking and acceptability study is in Figure S1.

### 2.5. Consumer Questionnaire

Volunteers were interviewed during screening at two locations in Auckland (AUT North Campus and surrounding area in Akoranga Northshore; AUT City Campus and surrounding area at Auckland city centre) using central location testing. Participants (*n* = 91) who regularly consume rice as a staple grain completed questionnaires to record demographics (age, gender, and ethnicity) and rice consumption habits (rice type, rice product, cooking method, frequency and the amount of rice consumed). Each participant completed a hard copy of the questionnaire. The data were entered into an Excel spreadsheet (Office 2015, Microsoft, Microsoft Corporation (MS), Washington, DC, USA) by the investigator after data collection was completed. The data were checked by an independent investigator.

### 2.6. Consumer Liking Testing and Acceptability

**Figure 1:**
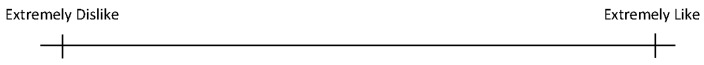
Visualised Analogue Scale (VAS) for measuring consumer liking in relation to sensory characteristics (colour, taste, texture, and overall acceptability) on a 100 mm unstructured line.

All participants (*n* = 91) who completed questionnaires were asked to attend the tasting session. Twenty-seven participants dropped out because of not fasting (*n* = 7) and unavailability (*n* = 20). A total of 64 participants completed the consumer liking and acceptability trial. Consumer liking testing was conducted at AUT North Campus, Auckland, New Zealand between the hours of 10:00 a.m. and 11:00 a.m. The six rice portions were prepared and subjected to effective testing (i.e., consumer liking) in accordance with Lawless and Heymann [10] using Visual Analogue Scales (VAS). Each rice portion (50 g) was assigned a 3-digit random code and presented unbranded under a clear food wrap cover. The six samples were assessed at the same time in individual booths under white light at room temperature (23 ± 2 °C) and humidity of 35% ± 3%. Each participant (*n* = 64) tasted the six samples in a blind condition and evaluated the liking of each rice sample in relation to the sensory characteristics (colour, taste, flavour, texture, and overall acceptability) on five 100 mm unstructured line VAS (Figure 1).

To reduce the first order and carryover effects, the order of sample presentation was randomised using Williams Latin square design [14]. Each participant was required to break for 2 min between each sample and cleanse their palate by rinsing their mouth with filtered room temperature water. 

In the second stage, all participants were presented with all six samples together and information of the rice type, cooking and preparation methods, and nutritional information (i.e., starch digestibility and glycaemic impact). The nutritional differences among the six rice samples are presented in Table S1. Each participant tasted the six samples and evaluated the overall acceptability of the six rice samples on six 100 mm unstructured VAS anchored at extremely dislike on the left end and extremely like on the right end (Figure 1). The question of overall acceptability was “Would you consider replacing your current rice meal with this rice sample?”

Each participant evaluated the liking and the degree of acceptability by using a pen to mark a vertical line on the VAS, which best represented the participant’s response at that time. Liking of the sensory properties and the degree of acceptability were hand-measured between the left hand anchor point and the marked point to the nearest mm. The length (mm) was then recorded and entered into an Excel spreadsheet (Office 2015, Microsoft) by the investigator. An independent investigator re-measured the length (mm) and checked the data. 

### 2.7. Data Analysis

Liking characteristics were compared using two-way repeated measures analysis of variance (ANOVA) with post-hoc Tukey’s honestly significant difference (HSD) tests. The six rice samples were entered as a fixed factor and participants as a random factor to determine the liking and acceptability that were discriminatory (*p* < 0.05) between rice samples. Data analysis was carried out using SPSS 12.0.1 (SPSS Inc., Chicago, IL, USA). 

Hierarchical clusters analysis (HCA) with squared Euclidean distance and Ward’s criterion was carried out using SPSS 12.0.1 (SPSS Inc.) to investigate the existence of homogeneous clusters of participants with similar overall acceptability for all six rice samples after being informed about the rice samples. For each separate cluster, overall acceptability was analysed using a repeated measures ANOVA (with post hoc HSD) test, with rice samples as a fixed factor and participant as a random factor. In addition, the participant clusters were compared in terms of demographic data using an approximate chi-square test for similarity among groups.

## 3. Results

### 3.1. Consumer Questionnaire

Ninety-one Auckland rice consumers completed the questionnaire and demographics at both study locations. Around 25% more females (*n* = 57) than males (*n* = 34), and approximately 27% more “Europeans and others” (*n* = 58) than “East Asians” (*n* = 33), were interviewed (Table 1). No significant differences in age and rice consumption habits (frequency and the amount of rice consumed per week) were observed by gender. The East Asian consumers were around 10 years younger than the European and others consumers (*F*-value = 11.346, *p*-value = 0.001). Average East Asian consumers ate three times more rice than Europeans and others per week (*F*-value = 68.587, *p*-value < 0.001). Around 30% more participants (in both genders and both ethnic groups) consumed refined or white rice than wholegrain or brown rice regularly. Almost half the participants reported commonly consuming freshly boiled or steamed rice, while significantly fewer participants consumed reheated rice. Around 57% East Asian consumers preferred freshly boiled or steamed rice while more than half of Europeans and others consumers preferred stir-fried rice. Generally, around 10% more participants preferred rice meals from restaurants or take-away stores.

### 3.2. Liking of the Sensory Characteristics

When the rice samples were presented without nutritional value information (i.e., rice variety, preparation method, and starch digestibility), the texture, flavour, and taste discriminated significantly among rice samples (Table 2). The average liking of colour was not significantly different among the six rice samples (*F* = 1.574, *p* = 0.167, eta2 = 0.003). Freshly cooked medium-grain brown, reheated parboiled, and reheated medium-grain brown rice samples scored similarly on overall liking, which was significantly higher than for freshly cooked white rice.

Reheated parboiled rice and freshly cooked medium-grain brown rice samples received higher liking scores on all four sensory characteristics compared to other rice samples. The liking scores for flavour and taste were similar between the two rice samples. However, participants reported higher liking of the texture of freshly cooked medium-grain brown rice than reheated parboiled rice, and higher liking of colour of reheated parboiled than freshly cooked medium-grain brown rice.

The cold storage and reheating treatment had a more significant effect on parboiled rice than other rice varieties. The post-cooking treatment significantly improved the liking of colour, flavour and taste of parboiled rice whilst reducing the texture. While the same post-cooking treatment significantly reduced texture scores and improved colour scores for medium-grain brown rice, there was a minimal effect on flavour and taste. Both reheated and freshly cooked medium-grain white rice samples had significantly lower scores for liking on the four sensory characteristics compared to other samples. The liking scores for flavour and taste were similar between these two medium grain white rice samples. However, the cold storage and reheating treatment reduced the colour and texture of medium grain white rice.

### 3.3. Acceptability

When participants were asked to rank the overall acceptability of consuming the rice sample as the replacement of the staple rice meal they commonly consume, freshly cooked medium-grain brown rice and the reheated parboiled rice were both ranked as acceptable. HCA identified three clusters of similar overall acceptability of the six rice samples. The three clusters consisted of 21.9% (*n* = 14), 21.9% (*n* = 14), and 56.3% (*n* = 36) of participants, respectively. For each cluster, ANOVA results showed that consumers significantly differentiated among the rice samples (Table 3). Participants tended to prefer the medium-grain brown rice and parboiled rice, both freshly cooked and reheated. However, cluster 2 participants tended to prefer freshly cooked rice samples, whilst cluster 3 participants preferred reheated ones. Participants in cluster 1 preferred the freshly cooked parboiled and medium-grain brown rice to reheated counterparts; however, they significantly favoured the reheated medium-grain white rice over other reheated samples.

Demographic characteristics and rice consumption habits were compared among three clusters (Table S2). Cluster 3 comprised two-thirds of the adults between 36 and 55 years, while participants in the other two clusters were much younger (18 to 35 years). Most participants in cluster 1 consumed rice meals less than 10 times per month (75%), while those in clusters 2 (79%) and 3 (84%) consumed more rice more than 10 times per month. Participants in clusters 1 and 3 were predominantly European and others (over 85%) and in cluster 2 were East Asian (78.2%). More participants in cluster 3 commonly ate both brown rice (58.2%) and white rice (41.8%) prepared at home (63.8%), while the other two clusters reported that they ate white rice (62.5% and 68.2%, respectively) at restaurants or from takeout (68.8% and 72.7% respectively). Cluster 1 can be characterised as younger Europeans who occasionally eat white rice at a restaurant or takeout; cluster 2 as younger Asian consumers who regularly eat white rice at a restaurant or takeout; and cluster 3, the largest cluster, as middle aged consumers from both ethnic groups who commonly consume both brown rice and white rice as part of a home-cooked meal.

## 4. Discussion

Overall, reheated parboiled rice was rated favourably in terms of colour, taste and flavour, and could be accepted as an alternative to freshly cooked or reheated medium-grain white rice. In addition to liking based on sensory characteristics, the favourable glycaemic properties of reheated parboiled rice [1,2] provides evidence that it could be recommended for a healthier diet. Previous studies have found an overall acceptability of rice of over 5.0 on average for freshly cooked rice, using a 10-point categorical Likert scale (1 = extremely dislike and 10 = extremely like) [7,15], which is consistent with the results of this study. The present study has demonstrated the feasibility of a longer-term dietary intervention involving consumption of parboiled rice and adoption of safe cold storage and reheating post-cooking treatment in a multi-ethnic population [2].

The overall acceptability ratings of parboiled rice and medium-grain brown rice were higher than medium-grain white rice samples when consumed freshly cooked or reheated. This trend was associated with the higher liking of all four characteristics (i.e., texture, flavour, taste, and colour) of both medium-grain brown rice and parboiled rice, in which rice sensory profiles are mostly formed during process-induced changes (i.e., polishing and parboiling pre-treatment) [8]. The higher total lipids deposition on the surface of brown rice bran (60% to 80% higher compared to polished white rice) undergoes lipase and subsequent oxidation and is hydrolysed to free fatty acids, producing a distinct colour and flavour [16]. Polyphenols in rice bran may also be associated with a bitter or astringent taste [17]. The bran residue increases the total dietary fibre content and gives the cooked brown rice a nutty texture [18]. Mixed rice acceptability ratings have been observed in previous studies. Muhihi, et al. [19] reported whole-grain brown rice as highly acceptable among overweight and obese Tanzanian adults in terms of smell, taste, colour, appearance, and texture. However, studies in Costa Rica [20], China [21], and South India [22] reported that local consumers preferred polished white rice and the major barriers for accepting whole-grain brown rice were its chewy and nutty texture, poor appearance (colour), and distinct flavour. Although no study has investigated consumers’ acceptability of whole-grain rice verses refined-grain rice in Western countries, a number of studies have reported that European consumers (in the United Kingdom, Italy, Finland, and Germany) favoured wholegrain cereal and wheat products [23,24] due to high awareness of the health-related benefits of wholegrain products. This is consistent with the present findings, which reported that New Zealand European participants preferred wholegrain to white rice, while East Asian participants preferred the opposite. 

After parboiling and polishing, parboiled rice loses the bran and crude fat content; however, soaking at high temperatures during parboiling makes parboiled rice retain its colouration, nutty chewy texture, and a distinct flavour [25]. Present findings are consistent with previous studies, which observed that white rice and parboiled rice samples presented comparable levels of appearance [7,26], and whiteness was less important in quality perception of rice products [7,27]. More recent studies in India by Sudha et al., [22] and Kumar et al. [28] also reported that parboiled rice was favoured by participants compared with brown rice because its appearance and aroma after polishing represented higher quality. The present study also reported that European participants, had a higher acceptability of parboiled rice compared to Asian participants. This could be associated with Europeans’ liking of nutty and pigmented whole-grain rice.

Cold storage and reheating preparation significantly improved participants’ liking of flavour and taste of parboiled rice. Liking of taste is significantly correlated with the liking of flavour. Decreased liking of taste and flavour might be due to reduced starch digestibility after cold storage and reheating (i.e., increased proportion of resistant starch and slowly digested starch) [1,2], with less oral hydrolysis and consequently decreased oral sugar release. Decreased liking of taste in rice also contributed to a healthier image of rice meals [26,28,29]. Previous studies in India [28,30] found that participants generally preferred grains that were less sweet with a less creamy flavour. However, studies in East Asia [21,31] found that participants preferred the creamier flavour in refined grains. As the present study’s participants consisted of around 25% of people of East Asian origin and 75% of European and South Asian origins, the increase in overall acceptability, liking of flavour and taste could be attributed to the differences in liking between ethnic groups from which the participants in the present study were drawn [32,33].

Cold storage and reheating only slightly improved the liking of the texture of medium-grain white rice, while it reduced the liking of the texture of parboiled and medium-grain brown rice. It is suggested that cold storage and reheating reduced the moisture content and increased the gelatinised starch recrystallization in medium-grain white rice, as was observed in previous in vitro studies of starch digestibility in rice [1], and might have reduced the grain adhesion and increased hardness, resulting in an increased liking of the texture [26] as the firmer texture was generally favoured by participants of European and South Asian origin [19,28,30]. However, the decrease in adhesion and softness during storage is higher for long-grain rice (i.e., high amylose parboiled rice) [8], in which cold storage may have increased firmness, resulting in reduced liking of the texture. Similarly, cold storage of whole-grain brown rice might have resulted in a significantly firmer texture with bran intact; therefore, it may result in a significantly firmer texture. 

Food habits and culture could play a significant role in accepting parboiled and brown rice products and the optimisation of reheating methods for some ethnic groups [7,19,21,27,29,34]. Studies in China [21] and Costa Rica [20] reported that participants perceived brown rice as a less accepted product in terms of taste, quality, family tradition, and social status. Kumar et al. [28] and Sudha et al. [22] also suggested that consumers tend to prefer the rice product that has been consumed by the family for generations. Similarly, Behrens et al. [34] and Heinemann [7] suggested the lack of knowledge of the nutritional aspects of parboiled rice and the unfamiliarity with parboiled rice could reduce its acceptability among rice consumers. A consumer’s prior experience with a product might influence the liking and acceptability of it [35]. The present study confirmed the hypothesis that participants who prepare and consume brown rice at home regularly (more than 10 times per month), preferred reheated brown rice and parboiled rice compared to the participants who consume white rice regularly. Acceptance of healthier rice choices may be improved by nutrition and health education of the potential health benefits and nutritional value (i.e., glycaemic lowering effect) of parboiled and brown rice, and knowledge of the method for cooking them [20,21,22]. 

However, neither nutritional information, nor knowledge of the reheating method alone are able to impact consumers’ acceptability of parboiled and brown rice. A recent review by Heiniö et al. [29] suggested that liking for the sensory characteristics (i.e., colour, odour, texture, and flavour) in refined grains could contribute to the lower acceptability of whole-grain cereals. The results of the present study, which compared sensory characteristics of reheated parboiled rice and other samples, support the claim that higher acceptability contributes to healthier and more sustainable diets.

The design and execution of the study followed the requirements for a reliable and credible laboratory-based sensory liking test [10], which was powered to detect minimal differences in the VAS ratings given for the rice samples [10,36]. The other advantage of this study is that the selection of participants was not designed to have an even number of participants in each age, gender and ethnic group, but the participant population may represent the diverse Auckland community who eat rice. All participants were asked to fast for at least two hours before testing and rinse their mouth thoroughly between testing of each sample, in order to avoid possible misjudging or bias. The other strength of this study is the novelty of the study design. No recent study has examined the effect of a home-prepared cold storage and reheating treatment on the sensory characteristics of rice (overall acceptability, colour, texture, flavour and taste). 

The main limitation of this study is that it compared medium-grain white, medium-grain brown and parboiled rice only once, with a relatively small number of participants. Previous studies have introduced a multi-sample repeated measure on one participant on separate days in order to minimise Type II error [10]. It is suggested that a repeated measure be introduced to test within-individual variance. In addition, only five sensory characteristics (colour, flavour, texture, tastes, and overall acceptability) were compared, and other factors such as mood and when last eaten, that may have influenced participants’ liking, were not measured. As this study was not designed to compare the age, gender and ethnic effect on liking, these factors were not compared. There may be a natural variation in liking among different demographic groups [10]. 

## 5. Conclusions

The findings of this study corroborate the need for marketing efforts that can effectively inform consumers about the nutritional and health advantages of prolonged cold treatment (cold storage of cooked rice products at 4 °C for more than 24 h) and reheating; the nutritional values; and convenience of parboiled rice. The cold-treated and reheated rice preparation method would be more likely to be accepted by European older adult consumers, who are increasingly preparing and consuming rice domestically. Compared to these rice consumers, those who are accustomed to consuming rice as a traditional food might prefer the freshly boiled preparation method. This information may contribute to increasing public awareness and, eventually, bring the nutritional benefits to different rice consumer populations. The messages may be particularly advantageous to people who are at risk of metabolic health issues, such as type 2 diabetes and gestational diabetes. Moreover, the results of the present study may support the potential opportunities of introducing pre-cooked plain rice products as a convenient and healthy staple meal.

## Figures and Tables

**Table 1:** Demographics of interviewed rice consumers (*n* = 91) at Auckland Akoranga (Northshore area) and city centre area.

Demographic Variables	Total (*n* = 91)	Gender		Ethnic Group ^1^	
		Male (*n* = 34, 37.4%)	Female (*n* = 57, 62.6%)	European and Others (*n* = 58, 63.7%)	East Asian (*n* = 33, 36.3%)
Average age (years, 95% CI)	38.9 (35.9, 41.9)	39.4 (35.1,43.8)	38.6 (34.5,42.7)	42.5 (38.5, 46.6)	32.6 (29.3, 35.9)
*Age group (%)*					
18–35 years	49.5%	41.2%	54.4%	37.9%	69.7%
36–55 years	36.3%	47.1%	29.8%	43.1%	30.3%
56 over	14.2%	11.7%	15.8%	19.0%	0%
Average time per month consumer eats rice (*n*, 95% CI))	19.0 (15.5, 22.5)	21.4 (15.9, 27.0)	17.6 (13.0,22.2)	10.7 (8.6,12.8)	33.6 (27.1,40.2)
*Times per month consumer eats rice (%)*					
4–10	37.4%	23.5%	45.6%	55.2%	6.1%
11–20	24.2%	26.5%	22.8%	29.3%	15.2%
20+	38.5%	50.0%	31.6%	5.5%	78.8%
Amount of cooked rice consumed per month (grams) ^2^	2850 (2330, 3380)	3210 (2380, 4050)	2640 (1950, 3330)	1610 (1300, 1910)	5050 (4060, 6030)
*Commonly consumed rice types (%)* ^3^					
Refined, white	65.9%	67.6%	64.9%	67.2%	63.6%
Wholegrain, brown	34.1%	32.4%	35.1%	32.8%	36.4%
Parboiled	0%	0%	0%	0%	0%
*Common Cooking method (%)*					
Boiled or steamed freshly	46.2 %	47.1%	46.6%	39.7%	57.6%
Stir-fried	38.5%	41.1%	36.8%	51.7%	15.2%
Boiled or steamed freshly and reheated ^4^	15.4%	11.8%	17.5%	8.6%	27.3%
*Where consumer prepare rice (%)*					
Home prepared	41.8%	38.2%	43.9%	39.7%	45.5%
Restaurant and take-away	58.2%	61.8%	56.1%	60.3%	54.5%

Note: ^1^ All ethnicities were self-identified. The “European and others” ethnic group includes New Zealand Pakeha, Maori, and Pacific ethnicities. Two Maori and three Pacific participants were interviewed. The East Asian ethnic group includes Chinese, Korean and Japanese people. ^2^ The amount of rice consumed each time was estimated by “cups of cooked rice consumed x estimated amount (g) per cup”. ^3^ Commonly consumed rice is defined as the rice that is consumed more than 50% of the time. ^4^ Reheated rice was described as cooked rice that has been stored for no more than 24 h and reheated before consumption.

**Table 2:** Participant (*n* = 64) liking scores (mm out of 100 mm) for colour, texture, flavour and taste, and overall acceptability of each cooked plain rice sample.

Rice Sample	Liking of the Sensory Characteristics ^1,2^ (mean (mm) (95% CI))			
	Colour	Texture	Flavour	Taste
Freshly cooked parboiled rice	59.1 (53.8, 63.1)	55.2 (49.8, 60.6)	50.6 (44.9, 56.3)^a^	48.8 (43.1, 54.6)^a^
Freshly cooked medium-grain brown rice	60.1 (55.0, 65.2)	58.0 (52.9, 63.1)^a^	59.2 (54.2, 64.2)^b^	50.9 (45.7, 56.2)^a^
Freshly cooked medium-grain white rice	59.1 (54.1, 64.2)	46.3 (40.0, 52.5)^b^	43.1 (37.5, 48.8)^a^	42.9 (37.1, 48.7)^b^
Reheated parboiled rice	61.3 (56.1, 66.4)	52.5 (46.3, 58.6)	57.2 (51.6, 62.8)^b^	54.3 (48.4, 60.2)^a^
Reheated medium-grain brown rice	60.9 (55.7, 66.0)	52.1 (46.0, 58.2)	56.8 (51.2, 62.4)^b^	53.9 (48.1, 59.8)^a^
Reheated medium-grain white rice	58.6 (54.4, 62.8)	47.8 (42.5, 53.1)^b^	45.3 (39.5, 51.1)^a^	42.0 (36.2, 47.7)^b^
Total	59.7 (57.5, 61.7)	52.0 (49.6, 54.3)	52.0 (49.7, 54.4)	48.8 (46.4, 51.2)

Note: ^1^ Liking score is presented as mean (mm) (lower 95% CI, upper 95% CI of the mean). The highest score is 100 mm. ^2^ Different letters (a, b) indicate significant differences (*p* < 0.05) between rice samples for the same characteristic.

**Table 3:** Mean Visual Analogue Scales (VAS) score (mean, 95% confidence intervals) of overall acceptability scores for each cluster, including overall mean acceptability.

Rice Sample	Overall Acceptability VAS (mm)^1,2^			
	Cluster 1 (*n* = 14)	Cluster 2 (*n* = 14)	Cluster 3 (*n* = 36)	Overall (*n* = 64)
Freshly cooked parboiled rice	74.7 (64.6, 84.8) ^a^	51.4 (42.8, 60.0) ^a^	40.5 (32.6, 48.4) ^a^	52.8 (47.1, 58.5)
Freshly cooked medium grain brown rice	61.8 (50.8, 72.9) ^bd^	56.0 (46.6, 65.4) ^a^	57.2 (48.5, 65.8) ^bc^	57.9 (52.2, 63.7)
Freshly cooked medium grain white rice	25.4 (14.3, 36.5) ^c^	55.8 (46.3, 65.2) ^a^	45.8 (37.1, 54.5) ^ac^	44.1 (38.4, 49.9)
Reheated parboiled rice	56.1 (47.9, 64.3) ^b^	34.7 (27.7, 41.7) ^b^	74.3 (67.9, 80.7) ^b^	56.2 (50.4, 61.9)
Reheated medium grain brown rice	55.7 (47.6, 63.9) ^b^	34.5 (27.6, 41.4) ^b^	73.8 (67.4, 80.2) ^d^	55.8 (50.0, 61.5)
Reheated medium grain white rice	64.7 (54.7, 74.6) ^abd^	39.9 (31.4, 48.4) ^b^	51.5 (43.6, 59.3) ^ac^	50.8 (45.1, 56.5)
F-value	18.83	6.48	10.42	2.97
*P*-value	<0.001	<0.001	<0.001	0.012

**Note:**^1^ Acceptability score is presented as mean (mm) (lower 95% CI, upper 95% CI of the mean). The highest score is 100 mm. ^2^ Values with different letters indicate that their mean values are significantly different (*p* < 0.05) in the same column among three clusters, tested by repeated measures ANOVA. Different letters (a, b, c, d) indicate significant differences between clusters (*p* < 0.05).

## Supplementary Materials

The following are available online at http://www.mdpi.com/2304-8158/7/12/207/s1, Figure S1: Flow chart of the experiment design for single blinded consumer liking and unblended consumer acceptability, Table S1: Comparison of the starch digestibility profile among six rice samples: medium grain white, medium brown, parboiled rice, either freshly cooked or prolonged cold-treated (at 4 °C for 24 h), Table S2: Comparison of the demographic characteristics and rice consumption habits among three clusters.
