# Supplementary material for: The Effect of Cold Treatment of Parboiled Rice with Lowered Glycaemic Potency on Consumer Liking and Acceptability
Source: Foods. 2018 Dec 16;7(12):207. doi: 10.3390/foods7120207 (PMC6306780; doi:10.3390/foods7120207)
Supplement: Supplementary file 1 [file foods-07-00207-s001.pdf]

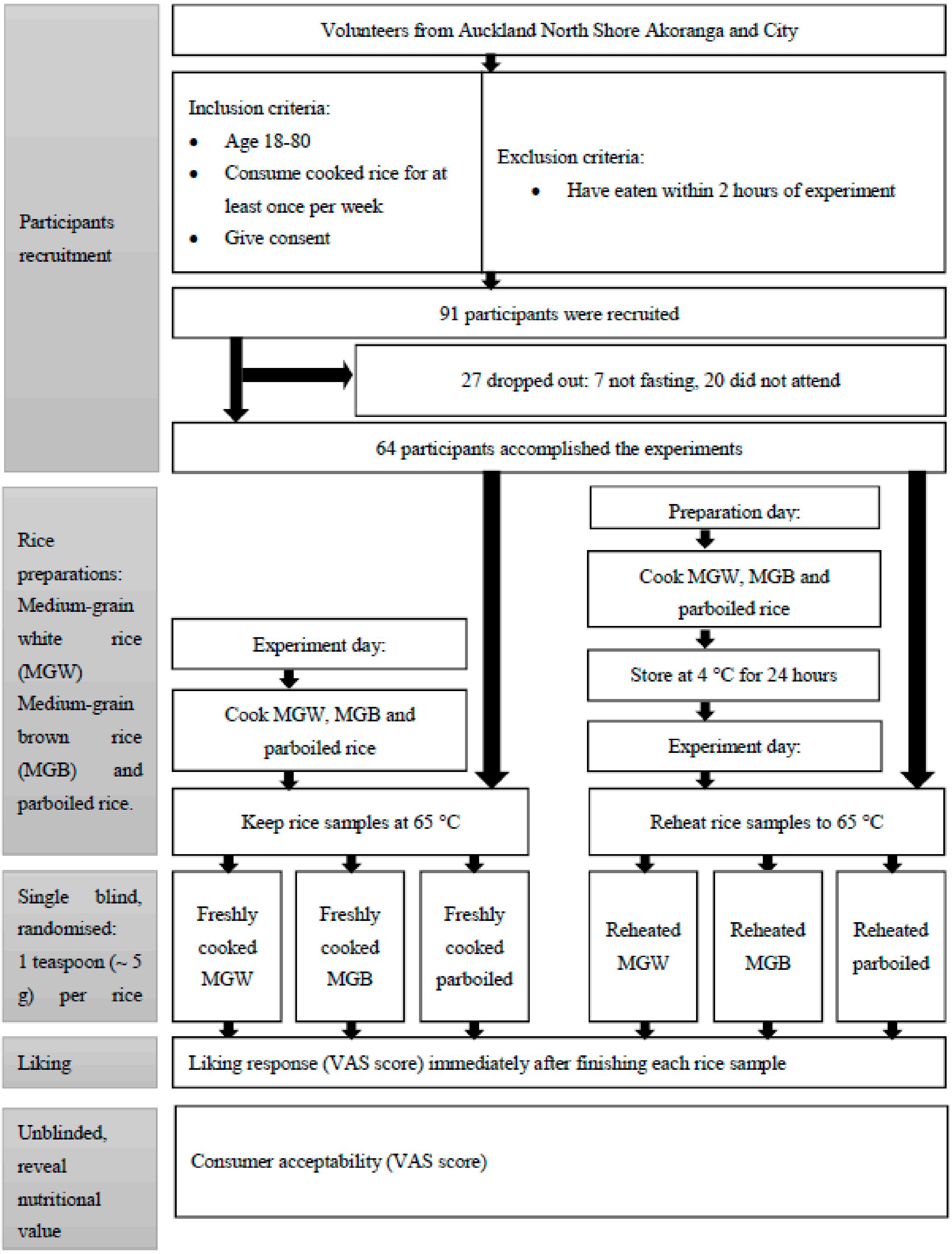

Figure S1. Flow chart showing the experimental design for single blind consumer liking and unblinded consumer acceptability.

Table S1. Comparison of the starch digestibility profile among six rice samples: Medium grain white, medium brown, parboiled rice, either freshly cooked or prolonged cold treated (at 4 °C for 24 h).

Rice sample	Preparation method	Total available starch (%)[1]	Resistant starch (%)[2]	Rapidly digestible starch (%)[3]	Slowly digestible starch (%)[4]
Medium grain white	Freshly cooked[5]	66.5 (6.2)	33.5 (6.2)	56.0 (4.5)	10.5 (1.7)
	Cold treated[6]	76.1 (1.6)	23.9 (1.6)	28.4 (0.7)	47.7 (2.3)
Medium grain brown	Freshly cooked	69.4 (2.3)	30.6 (2.3)	61.1 (4.2)	8.3 (2.0)
	Cold treated	76.0 (5.0)	24.0 (5.0)	41.8 (1.0)	34.2 (4.0)
Parboiled	Freshly cooked	71.6 (2.2)	28.4 (2.2)	42.2 (0.9)	29.3 (1.3)
	Cold treated	34.7 (1.0)	65.3 (1.0)	11.6 (0.2)	23.1 (1.2)

Note: [1] The total available starch (TAS) was determined as the proportion of the starch that can be digested within 180 minutes of consumption. TAS (%) = starch digested within 180 min (g/100 g rice dry weight basis) / total starch (TS) (g/100 g rice dry weight basis) * 100%; [2] the resistant starch (RS) was determined as the proportion of starch that cannot be digested after 180 minutes following consumption. RS (%) = (TS (g/100 g rice dry weight basis) – TAS (g/100 g rice dry weight basis)) / TS (g/100 g rice dry weight basis) * 100%; [3] the rapidly digestible starch (RDS) was determined as the proportion of starch that can be digested within 20 minutes of consumption. RDS (%) = starch digested within 20 min (g/100 g rice dry weight basis) / TS (g/100 g rice dry weight basis) * 100%; [4] the slowly digestible starch (SDS) was determined as the proportion of the starch that can be digested between 20 and 180 minutes of consumption. SDS (%) = (TAS (g/100 g rice dry weight basis) – RDS (g/100 g rice dry weight basis)) / TS (g/100 g rice dry weight basis) * 100%; [5] freshly cooked preparation method: Rice sample was freshly boiled as described in section 2.2. [6] Cold treated preparation method: Rice was cold treated at 4 °C for 24 h, as described in section 2.2.

Table S2. Comparison of the demographic characteristics and rice consumption habits among three clusters.

Demographic variables	Cluster 1 (n=14)	Cluster 2 (n=14)	Cluster 3 (n=36)
Age group (%)			
18–35 years	43.8	40.9	22.2
36–55 years	50.0	36.4	66.7
56 years over	6.2	22.7	11.1
Ethnic group (%)			
European and others	85.6	21.8	87.0
East Asian	14.4	78.2	13.0
Rice consumption (%)			
White rice	62.5	68.2	41.8
Brown rice	37.5	31.8	58.2
Times per month rice consumption (%)			
4 to 10	75.0	21.0	16.0
over 10	25.0	79.0	84.0
Where consumer prepare rice (%)			
Home prepared	31.2	27.3	63.8
Restaurant and take-away	68.8	72.7	36.2
